# Host association and selection on salivary protein genes in bed bugs and related blood-feeding ectoparasites

**DOI:** 10.1098/rsos.170446

**Published:** 2017-06-21

**Authors:** Benoit Talbot, Ondřej Balvín, Maarten J. Vonhof, Hugh G. Broders, Brock Fenton, Nusha Keyghobadi

**Affiliations:** 1 Department of Biology, University of Western Ontario, 1151 Richmond Street, London, Ontario, CanadaN6A 3K7; 2 Department of Ecology, Czech University of Life Sciences Prague, Kamýcká 129, 165 00 Praha 6 - Suchdol, Czech Republic; 3 Department of Biological Sciences, Western Michigan University, 1903 W Michigan Avenue, Kalamazoo, MI 49008-5410, USA; 4 Department of Biology, University of Waterloo, 200 University Avenue West, Waterloo, Ontario, CanadaN2L 3G1

**Keywords:** apyrase, candidate genes, coagulation, nitrophorin, phylogenetics

## Abstract

Reciprocal selective pressures can drive coevolutionary changes in parasites and hosts, and result in parasites that are highly specialized to their hosts. Selection and host co-adaptation are better understood in endoparasites than in ectoparasites, whose life cycles may be more loosely linked to that of their hosts. Blood-feeding ectoparasites use salivary proteins to prevent haemostasis in the host, and maximize energy intake. Here we looked for signals of selection in salivary protein genes of ectoparasite species from a single genus (*Cimex*) that associate with a range of hosts including mammals (bats and humans) and birds (swallows). We analysed two genes that code for salivary proteins that inhibit platelet aggregation and vasoconstriction and may directly affect the efficiency of blood feeding in these species. Significant positive selection was detected at five codons in one gene in all bat-associated species groups. Our results suggest association with bats, versus humans or swallows, has posed a selective pressure on the salivary apyrase gene in species of *Cimex*.

## 1. Background

Selection pressures imposed by antagonistic interactions between species, such as predators and prey or parasites and hosts, can produce evolutionary ‘arms races’ and are important drivers of adaptation and diversification [1–4]. Among parasites and their hosts, parasite species may evolve phenotypes that are increasingly efficient at using host resources. Hosts evolve phenotypes that are increasingly efficient at guarding against the loss of such resources. Such reciprocal evolutionary interactions may lead to parasite species becoming increasingly specialized to their host species, and to rapid evolution in genes involved in mediating the conflict [5,6].

Among parasite species, there is considerable variation in the extent to which the parasite individual is dependent upon and tied to a host individual. For example, a parasite may spend only part (temporary parasites), as opposed to all (permanent parasites), of its life cycle associated with its host [7,8]. Also, parasites may occur within the host's body (endoparasite) or on the outside (ectoparasite) [7,8]. Evolutionary arms races should be especially intense in permanent parasites and endoparasites, which are also often highly specialized [2,9]. Although temporary parasites and ectoparasites are likely to be more generalist and associate with more than one host species [10], possibly due to being more likely to encounter an alternative host, specialization and adaptation to a host can still occur in such parasites. For example, bat flies are temporary blood-feeding ectoparasites of bats, and yet they show very narrow host ranges [11]. Genetic adaptation to the host has been best studied in species that are tightly linked to their host, such as permanent parasites or endoparasites [2,3,12], but has not been extensively examined in temporary parasites or ectoparasites (but see [13,14]).

Adaptation of parasites to their specific hosts may be reflected in patterns of variation in parasite genes that are involved in mediating the host–parasite conflict, as suggested by Mans *et al.* [14] and Arcà *et al.* [15]. Specifically, host adaptation is suggested when non-synonymous substitutions at such genes, among parasites associating with different hosts, are more frequent than expected under neutral evolution (i.e. positive selection). By contrast, non-synonymous substitutions that are less frequent than expected under neutral evolution indicate selection for the conservation of gene sequences and function (i.e. negative selection). Here, we determined whether there is evidence of positive selection and host adaptation in a group of temporary ectoparasite species (genus *Cimex*, order Hemiptera) that associate with bats, humans or swallows, and that include a widespread human pest, the bed bug (*Cimex lectularis*).

Insects in the genus *Cimex* are temporary haematophagous ectoparasites of birds and mammals. They do not remain on their host at all times but rather stay in nests or roosts between blood meals [16]. Most *Cimex* species are associated exclusively with bats while a few associate mainly with humans and a few others with swallows [16–18]. Bats are hypothesized as ancestral hosts of the genus, and a move to human or bird hosts may have occurred when these cohabited in the same environments as bats, e.g. in caves [16,19]. The genus is traditionally divided into four species groups [16] whose identity was recently confirmed based on DNA analyses [20]. Members of the *Pilosellus* species group (represented by *Cimex adjunctus*, *Cimex brevis* and *Cimex latipennis* in this study), associate mainly with bats and occur in North America [16], while members of the *Pipistrelli* species group (e.g. *Cimex pipistrelli* or *Cimex japonicus*) associate mainly with bats in the Palaearctic region. Among the species that associate with swallows represented in the study, which are phylogenetically related to the *Pipistrelli* species group [20], *Cimex vicarius* occurs in North America, whereas *Cimex hirundinis* occurs in Europe [16], and a third currently unnamed species (*Cimex* sp*.*) occurs in Japan [20]. Finally, members of the *Lectularius* and the *Hemipterus* species groups are represented by the cosmopolitan bed bug (*C. lectularius*) and the tropical bed bug (*Cimex hemipterus*), which both have created specific host lineages associated with humans [16].

Studies of mRNA and proteins expressed in the salivary glands of *C. lectularius* [21–23] provide insights into how certain salivary proteins in this species act to suppress host defences (coagulation and vasoconstriction) at the site of rupture of a blood vessel, where the ectoparasite feeds. The anti-platelet property of apyrases results from catabolizing ADP released from damaged tissues. Apyrases in the saliva of *C. lectularius* could be used by the parasite to prevent clotting [21,23]. Nitrophorins, in the saliva of *C. lectularius*, have vasodilatory and anti-platelet property by transporting nitric oxide from the salivary glands to the feeding site [22,23]. Similar salivary proteins have been observed to be under positive selection in other blood-feeding arthropods, such as in mosquitoes [15], and potentially also in soft ticks [14] (but not in others, such as sandflies [13]), and therefore may play an important role in parasite adaptation to feeding on different host taxa.

We looked for evidence of positive selection acting on two salivary protein gene fragments, one coding for an apyrase and one coding for a nitrophorin, among *Cimex* specimens associated with bats, humans or swallows. We hypothesized that positive selection acts on those candidate genes, due to adaptation of species or lineages to blood feeding on phylogenetically different hosts. We therefore predicted codons of the two candidate genes would show significant signals of positive selection in most specimens. We also predicted that since association with humans and birds is hypothesized to have appeared after association with bats, specimens associated with humans or birds would show significant signals of positive selection at more codons that specimens associated with bats. We therefore predicted a significant difference in the number of codons showing signals of significant positive selection between specimens that are associated with different types of hosts (bats, humans or swallows).

## 2. Material and methods

### 2.1. Specimen collection

We processed whole body samples, stored in 95% EtOH, of cimicid ectoparasites, and also used sequence data from previous studies [20,24]. All cimicid samples were collected from the body of one of various bat and swallow species, from a roost mainly inhabited by one of various bat or swallow species or from human dwellings (electronic supplementary material, table S1). We analysed specimens from species from the four main *Cimex* clades (*Lectularius*, *Pilosellus*, *Hemipterus* and *Pipistrelli* species groups; [20]), as well as species associated with birds related to species in the *Pipistrelli* group. We identified individual samples to species using a combination of morphology [16] and DNA barcoding [25], where we compared the Cytochrome Oxidase 1 (*CO1*) sequence from each sample to known *CO1* sequences for *Cimex* species from published sources [20].

### 2.2. Genetic analyses

We extracted DNA from whole body samples using the DNeasy Blood & Tissue Kit (QIAGEN, Germantown, MD, USA). We then amplified fragments of the mitochondrial *CO1* gene and the nuclear Elongation Factor 1α (*EF1α*) gene using primers listed in the electronic supplementary material, table S2 [20,24,26]. We used these loci to construct an independent phylogeny of the specimens used in our study, to account for expected phylogenetic variances in our analyses of salivary protein genes. We also included published sequences (electronic supplementary material, table S1) of 11 *Cimex* specimens for one or both of the *CO1* and *EF1α* genes.

We designed primers to amplify fragments of two salivary protein genes (apyrase and nitrophorin) that have a known function that is directly linked to efficiency of blood feeding in *C. lectularius*, based on published mRNA sequences initially obtained from salivary glands of *C. lectularius* [21–23]. We designed our primers to maximize the size of the resulting fragment while also maximizing the number of specimens for which we obtained successful amplification. Our apyrase primers amplified a genomic fragment more than a third (371 bp) of the entire coding sequence (969 bp; [27]), that does not contain introns. Our nitrophorin primers also amplify a fragment more than a third (300 bp) of the entire coding sequence (840 bp; Protein Data Bank identifier: 1NTF [28,29]), which also does not contain introns. Resulting fragments code for a diversity of structural elements in both proteins, and encompass areas in the interior and exterior of the three-dimensional structure of the proteins [27,28].

We used a DNAEngine PTC-200 Thermal Cycler (BIO-RAD, Hercules, CA, USA) for Polymerase Chain Reaction (PCR) amplification for *CO1*, *EF1α*, and the apyrase and nitrophorin genes. For all gene fragments, we performed PCR in 25 µl final volume containing: 1X Taq Polymerase Buffer excluding MgCl_2_ (Applied Biosystems, Foster City, CA, USA), 1.5 mM of MgCl_2_, 0.2 mM of each type of dNTP, 0.3 µM of each primer, 1 U of Taq polymerase (ABI), and 2 µl of DNA extraction product. We used the following PCR cycling: an initial denaturation of 1 min at 94°C, followed by 35 cycles of 30 s of denaturation at 94°C, 45 s of annealing at a locus-specific temperature (42–57°C; electronic supplementary material, table S2) and 45 s of extension at 72°C, finished by a final extension step of 5 min at 72°C. We visualized PCR products by 1.5% agarose gel electrophoresis using SYBR Green stain (BIO-RAD) on a UV transluminator to check the quality and size of amplified fragments. Then we sequenced the amplified gene fragment for every sample using Sanger sequencing with BigDye terminator chemistry (ABI) and analysed the fragments on a 3730xl DNA Analyzer (ABI). We aligned all sequences using the MUSCLE function in MEGA 6.06 [30], and recorded heterozygous sites as ambiguous (N, which can be any of the four nucleotides).

### 2.3. Statistical analyses

We first constructed a hypothesized species tree, using information from both the *CO1* and the *EF1α* gene fragments, using the *BEAST framework in BEAST v. 2.4.2 [31]. The purpose was to obtain a phylogenetic tree showing the branch lengths (a proxy of branch-specific substitution rates) for all specimens. We used this phylogenetic tree in our analyses of selection (see following paragraphs) as an internal negative control [32]. This procedure maximizes the robustness of inferences about selection in candidate gene sequences (here, apyrase and nitrophorin genes) of specimens from a variety of species. To build a species tree, we used parameters recommended by the authors [33] to create 10 000 species trees using data from both loci: HKY substitution model with empirical allele frequencies, linear population size with constant root, 10 000 000 chains and storing every 5000th chain. We used a relaxed lognormal clock because it was shown to perform better than strict clock when substitution rates are expected to vary among lineages [34]. We then computed the maximum credibility tree [35] out of all the species trees produced by *BEAST, using TreeAnnotator in BEAST v. 2.4.2. We discarded the first 20% of Markov Chain Monte Carlo as burn-in, set no posterior probability limit, and set node heights at common ancestors.

Second, we found the substitution model best representing each candidate gene, using a function implemented in the web interface of the Hyphy 2.2.1 package [32]. We also performed a recombination detection analysis, using the Single Breakpoint Recombination (SBP; [36]) analysis in the web interface of the Hyphy 2.2.1 package, to see if our alignments showed any sign of recombination, which could bias our analyses of signals of selection. We used Beta-Gamma site-to-site rate variation and three rate classes, as these options are the most general with the fewest number of parameters, and should be realistic in most situations [32].

Third, as a prior assessment of whether any of the candidate genes shows a whole-sequence signal of positive selection, we performed a Partitioning Approach for Robust Inference of Selection (PARRIS) analysis [37], using the web interface of the Hyphy 2.2.1 package. This analysis compares a null model (without positive selection) and an alternative model (with positive selection) on the candidate gene sequences, using a likelihood ratio test.

Fourth, we used a suite of analyses to test if any codon in the candidate gene sequences is, or has been, affected by selection at any point in time. We used multiple analyses and considered only signals simultaneously supported by all analyses, to reduce the likelihood of false-positive results. We used three different likelihood-based approaches, implemented in the web interface of the Hyphy 2.2.1 package. Random-effects likelihood (REL; [38]) computes the likelihood that the ratio of non-synonymous to synonymous substitution rates at each codon fits with one of two predefined distributions, representing positive and negative selection, respectively, using information from the hypothesized species tree. Fixed-effects likelihood (FEL; [38]) compares the ratio of non-synonymous to synonymous substitution rates at each codon with the global expected substitution rates calculated using information from the hypothesized species tree. Mixed-effects model of evolution (MEME; [39]) compares the ratio of non-synonymous to synonymous substitution rates at each codon site with expected substitution rates specific to each node of the hypothesized species tree. All three methods can be used to detect codons under positive selection, but only REL and FEL are also aimed at detecting codons under negative selection. To reduce the possibility of false discovery, we considered only codons that were detected to be under positive selection by all three analyses (*α* < 0.05 for the MEME and the FEL approaches, or Bayes factor >100 for the REL approach), or under negative selection by both REL and FEL. Additionally, we used the empirical Bayes procedure implemented in the MEME approach (Bayes Factor >100) to identify nodes of the hypothesized species tree where a signal of significant positive selection is observed, at each codon previously identified as being under positive selection simultaneously by the REL, FEL and MEME approaches. The purpose was to determine which specimens, or groups of specimens, are characterized by a signal of positive selection at each of those codons.

Finally, we analysed the effect of association with one of three types of host (bat, human and swallow), by each specimen, on the number of codons showing signals of positive selection with all three approaches, using a standard analysis of variance function in R v. 3.2.2 [40]. We corrected the response and the predictor variables for phylogenetic independent contrasts, to account for shared history in each pair of specimens of the study. We built independent contrasts using our hypothesized species tree, in Newick format [41], with the ‘pic’ function from the ‘ape’ package [42] in R v. 3.2.2. This procedure maximizes the robustness of our correlative inferences, because it takes into account phylogenetic distances between specimens.

## 3. Results

We amplified and sequenced the target fragments for both salivary protein candidate genes, *CO1* and *EF1α* for 26 specimens of ten congeneric species (although three specimens did not amplify at the nitrophorin gene fragment; electronic supplementary material, table S1). Sequence lengths in the apyrase fragment varied by 66 bp, possibly due to deletion or insertion events. Although these indels possibly represent adaptive change, there is currently no way to test selection associated with them. We trimmed the start and the end of each sequence for both salivary protein genes, so that they only included whole codons, resulting in an alignment of 297 bp for the nitrophorin fragment and an alignment of 369 bp for the apyrase fragment. Up to 1% and 4% of the apyrase and nitrophorin gene sequences, respectively, in any individual, were heterozygous. For all analyses, we treated gaps and heterozygous loci as missing data, which was the most conservative option.

### 3.1. Species tree

**Figure 1.**
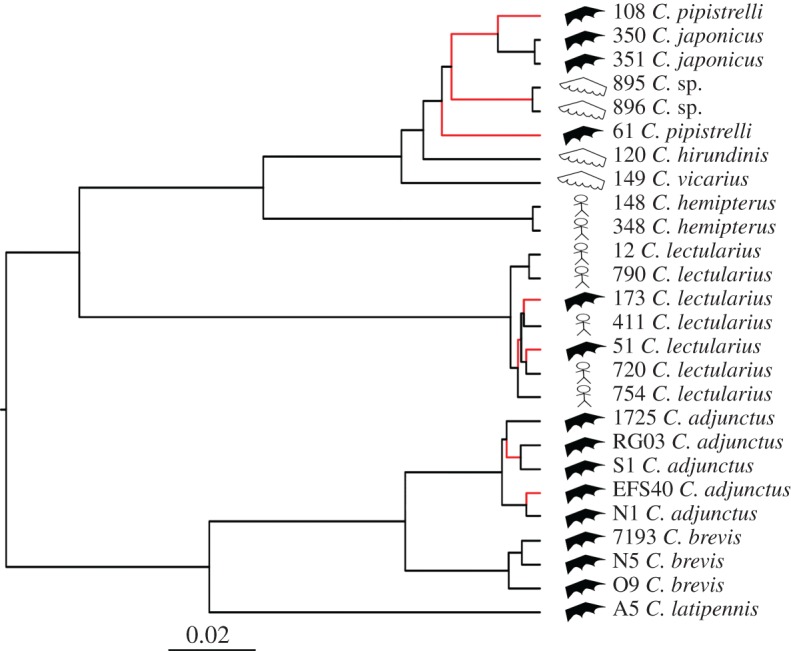
Hypothesized species tree of *Cimex* spp*.* specimens, based on *CO1* and *EF1α*, constructed with *BEAST 2.4.2. Scale represents substitutions per site. The specimen number (as in the electronic supplementary material, table S1), the species name and the host to which it was associated (stick figure for human, black pointed wing for bat and white rounded wing for swallow) of each sample are shown. Red-coloured branches indicate that significant positive selection was detected in the apyrase gene sequence of the corresponding specimen(s) at the terminal node, or at an internal node downstream from it (Bayes Factor > 100; calculated using the MEME approach), at any of five codons (37, 43, 63, 83, 93; previously identified as being under positive selection using the MEME, FEL and REL approaches).

As in Balvín *et al.* [20], our species tree, based on *CO1* and *EF1α*, shows clear distinctions among most species (figure 1; except for *C. pipistrelli*, as in Balvín *et al.* [20]). One clade representing the *Pilosellus* group contains three bat-associated species from North America (*C. adjunctus*, *C. brevis* and *C. latipennis*), one clade representing the *Lectularius* group contains only the cosmopolitan *C. lectularius*, one clade representing the *Hemipterus* group contains only the tropical human-associated *C. hemipterus*, and a final clade contains species mainly associated with bats or swallows, in North America, Europe and Asia (*C. pipistrelli*, *C. japonicus*, *C. vicarius*, *C. hirundinis* and *C.* sp).

### 3.2. Signals of selection on the apyrase gene

The best substitution model for the apyrase gene fragment specified a similar rate of evolution between both types of transitions and A to C (or C to A) transversions, and a different rate of evolution for all other types of transversions. Using the SBP approach, all three criteria (AIC, cAIC and BIC) gave best support for no recombination in the dataset.

Using the PARRIS approach to detect sequence-wide positive selection, we found a slightly higher log-likelihood value for the alternative model of positive selection (log (L) = −1718.5 for the null model; log (L) = −1714. 3 for the alternative model), leading to a likelihood ratio test value of 8.331 and a *p* of 0.016. The significant *p*-value suggests evidence of positive selection in the dataset.

**Figure 2.**
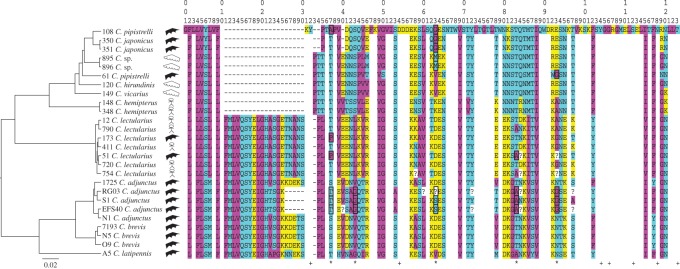
Translated (from DNA sequence) amino acid sequence of an apyrase gene for each cimicid ectoparasite sample in this study. Codon numbers (top rows) correspond to those in table 1 and electronic supplementary material, figure S1. Colour represents the chemical properties of amino acids (purple, non-polar; blue, polar uncharged; yellow, charged). Blanks refer to codon positions with no variation, hyphens refer to gaps in the alignments, question marks refer to codons containing heterozygous sites, asterisks refer to codons inferred to be under positive selection, using MEME, FEL and REL approaches, and pluses refer to codons inferred to be under negative selection, using FEL and REL approaches. A hypothesized species tree, based on *CO1* and *EF1α*, constructed with *BEAST 2.4.2 (scale represents substitutions per site), the specimen number (as in the electronic supplementary material, table S1), the species name and the host to which it was associated (stick figure for human, black pointed wing for bat and white rounded wing for swallow) of each sample is shown. Black rectangles around codon positions refer to detection of a significant signal of positive selection at the specific terminal node or at an internal node downstream from it, in the hypothesized species tree, using the MEME approach.

The FEL approach identified six codons under positive selection, whereas the MEME and REL approaches identified eight and 15, respectively. Five codons were identified by all three approaches and therefore have strong support for being under positive selection (table 1). The MEME approach suggests almost all evidence of positive selection in the five codons is in bat-associated lineages, and only one signal is in a swallow-associated lineage (figure 1; electronic supplementary material, figure S1 for results specific to each of the five codons). Furthermore, we found a significant effect (d.f. = 1, *F* = 4.678, *p* = 0.041) of association with a particular type of host on the number of codons showing signals of positive selection. Specimens associated with bats had on average more codons showing signals of positive selection than specimens associated with humans or swallows (mean ± variance: bat = 1.7 ± 3.8; human = 0.3 ± 0.2; swallow = 0.5 ± 0.3). Observed substitutions at the five identified codons are likely to cause major structural changes to the apyrase protein, because they are all characterized by switches between charged, or polar, amino acids and non-polar amino acids (figure 2). Also, observed substitutions at three of the five identified codons (43, 63 and 93) represent changes between small and large amino acids. Amino acid substitutions at the five codons inferred to be under positive selection are, however, different among specimens showing signals of positive selection.

**Table 1.** Significant tests of selection on fragments of two genes coding for salivary proteins, apyrase and nitrophorin, in blood-feeding cimicid ectoparasites. The test value (*p* or Bayes Factor) is given for all three codon-based analyses of positive and negative selection (MEME, FEL and REL), for each codon identified by all relevant analyses as showing a significant signal of selection. Codon numbers correspond to those used in figures 2 and 3 and electronic supplementary material, figure S1.

gene fragment	type of selection	codon number	MEME (*p*)	FEL (*p*)	REL (Bayes Factor)
apyrase	positive	37	0.027	0.035	751.9
		43	0.036	0.014	1860.5
		63	0.013	0.011	169142.0
		83	0.034	0.022	1162.7
		93	0.037	0.040	198.4
	negative	32	n.a.	0.001	432.7
		54		0.007	145.4
		104		<0.001	479.0
		106		0.007	283.6
		112		0.005	248.3
		118		<0.001	4908.9
		123		<0.001	3001.6
nitrophorin	negative	1	n.a.	0.007	875.2
		25		0.022	442.8
		32		<0.001	675630.0
		37		0.021	2605.2
		40		0.020	484.9
		55		0.007	394.1
		69		0.005	13861.0
		85		0.002	968.9
		88		0.003	212.1
		95		0.003	18721.4
		99		0.005	12754.0

The REL approach identified seven codons as potentially under negative selection, whereas the FEL approach identified the same seven codons, plus four additional ones. Therefore, seven codons have strong support for being under negative selection (table 1; MEME cannot detect loci under negative selection). All seven codons show no variation in amino acid, and two of the identified codons (54 and 112) are binding sites for calcium and nucleotides, respectively [27].

### 3.3. Signals of selection on the nitrophorin gene

The best substitution model for the nitrophorin gene fragment specified a similar rate of evolution in all types of transitions and transversions, a model best known as F81 [43]. Using the SBP approach, all three criteria (AIC, cAIC and BIC) gave best support for no recombination in the dataset.

Using the PARRIS approach to detect sequence-wide positive selection, we found similar log-likelihood values for the two models (log (L) = −1073.0 for the null model; log (L) = −1072.8 for the alternative model), leading to a likelihood ratio test value of 0.545 and a *p* of 0.761. The non-significant *p*-value suggests no evidence of positive selection in the dataset.

**Figure 3.**
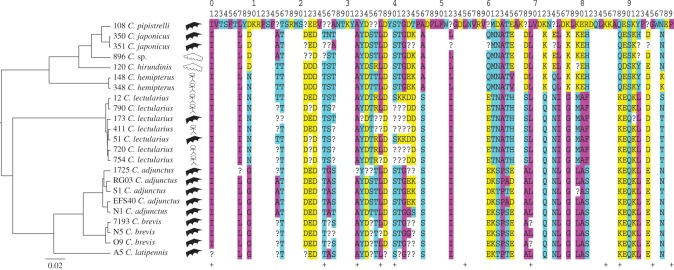
Translated (from DNA sequence) amino acid sequence of a nitrophorin gene for each cimicid ectoparasite sample in this study. Codon numbers (top rows) correspond to those in table 1. Colour represents the chemical properties of amino acids (purple, non-polar; blue, polar uncharged; yellow, charged). Blanks refer to codon positions with no variation, hyphens refer to gaps in the alignments, question marks refer to codons containing heterozygous sites, and pluses refer to codons inferred to be under negative selection, using FEL and REL approaches. A hypothesized species tree, based on *CO1* and *EF1α*, constructed with *BEAST 2.4.2 (scale represents substitutions per site), the specimen number (as in the electronic supplementary material, table S1), the species name and the host to which it was associated (stick figure for human, black pointed wing for bat and white rounded wing for swallow) of each sample is shown.

The MEME approach identified three codons under positive selection, as opposed to one each in the FEL and the REL approaches. However, no single codon was identified by all three analyses. The REL approach identified 11 codons potentially under negative selection and the FEL approach identified the same 11 codons, plus three additional ones. Therefore, no codon has strong support for being under positive selection and 11 codons have strong support for being under negative selection. Ten of the 11 codons inferred as being under negative selection show no variation in amino acid (Codon 88 shows variation between a polar and a charged amino acid across specimens, figure 3).

## 4. Discussion

### 4.1. Signals of selection on the apyrase and nitrophorin genes

We found signals of positive selection in the gene sequence coding for the salivary protein apyrase, among lineages of blood-feeding ectoparasites of the genus *Cimex*. A second candidate gene for the salivary protein nitrophorin did not show any signals of positive selection. We also found that the type of host (bats, humans or swallows) was correlated with the number of codons showing signals of positive selection at apyrase. However, contrary to our expectation, signals of positive selection were more frequent in bat-associated specimens than in swallow- or human-associated specimens. Our results indicate that association with bats has resulted in selective pressure on the gene coding for a salivary apyrase, a gene that is involved in preventing haemostasis at the feeding site in the host.

Host body temperature may be one possible factor that could result in divergent selection pressures on ectoparasites feeding on blood of bats, versus humans or swallows. We collected bat-associated individuals mostly on bats of Vespertilionidae, a diverse clade of insectivorous bats. Heterothermy is very common in these bats, and individuals frequently go into short bouts of torpor during which body temperature may drop rapidly [44]. Blood temperature of humans, on the other hand, is very stable, staying at around 37°C [45]. Blood temperature in barn swallows is also stable at around 41°C [46]. DeVries *et al.* [47] suggested host body temperature (simulated with a temperature-controlled feeder) may affect the blood-feeding behaviour of *C. lectularius*, where optimal temperatures ranged between 38 and 43°C. Therefore, apyrase in bat-associated taxa may be, or have been, affected by selective pressure from the relatively large daily temperature variation undergone in blood vessels of their bat hosts. However, this remains to be tested, by measuring the effect of amino acid substitutions on variation in three-dimensional structure of cimicid salivary apyrase, and by measuring the efficiency of several salivary protein variants at different temperatures.

On average, the ratio of the volume of red blood cells to the total volume of blood (i.e. haematocrit) in birds and bats is higher than in humans (59% in bat species [48] and 55.9% in barn swallows in Europe [49]; versus 42% in humans [48]). Blood cells contain the ATP-rich haem complex, and ATP was shown to be the most effective phagostimulant in *C. lectularius* [50], suggesting it is the main source of energy sought by *C. lectularius* while feeding. Ectoparasites feeding on humans may therefore need blood to flow for longer, compared to ectoparasites feeding on bats or swallows, to gain a similar amount of energy. As a result, one might expect selection on human-associated cimicids to prevent haemostasis more effectively. However, we did not see strong evidence for this as we obtained only one significant signal of positive selection in one lineage of human-associated cimicids in the apyrase gene. This lineage also includes two specimens from bat-associated *C. lectularius* populations, which were both found to possess an additional putatively advantageous substitution. Our results suggest lower haematocrit may not pose an important selective pressure on salivary apyrase or nitrophorin in cimicids feeding on humans, although it may pose a selective pressure on other genes not looked at in this study, or the effects of lower haematocrit may be weak compared with those of variation in body temperature. Additionally, a decrease in overall genetic diversity in human-associated cimicid populations potentially caused by recent founding events, as suggested by Booth *et al.* [51], could also dampen any signal of selection on salivary protein genes in those populations.

We observed significant signals of negative selection on fragments of both the apyrase and nitrophorin genes. Less than a third of codons identified as under negative selection in the apyrase fragment are active sites binding to calcium (a promoter) and to nucleotides (where ADP is hydrolysed; [27]). Codons displaying signals of negative selection in both proteins are located in the interior and exterior of the three-dimensional structure of the proteins, suggesting selection affects the whole protein structure, and is not limited to the active sites [27,28]. As expected, variation is very low at these specific codons in the two salivary protein genes, as changes in amino acid at those codons are presumably unfavourable. However, it is currently unclear how specific changes in amino acid at those codons would affect the function of the enzymes.

### 4.2. Species tree

Bats have been hypothesized to be the ancestral host of species of *Cimex*, and association to birds and humans putatively appeared subsequently [16]. One interesting finding from our study is that most bat-associated specimens show one or more signals of selection in a salivary protein gene, which is associated with feeding for these organisms, and almost none of the human-associated specimens show signals of positive selection. If bats are the ancestral host of the group, one might expect selection on salivary proteins to operate more strongly on specimens associated with different hosts than bats. For example, persistence of a strain of the rabies virus in a new host (from a bat species, which is thought to be the original host of rabies, to a canine species), requires multiple adaptive changes pertaining to replication and transmission [52]. However, it is important to note that our study was performed on only two genes, and analysing genes coding for other functional traits related to host use are essential to fully understand adaptation of cimicids to their hosts. Our study nonetheless is an important first step in an investigation of signals of positive selection in salivary protein gene sequences in this diverse and cosmopolitan group of ectoparasites. Our study also provides evidence of positive selection linked with association to a specific type of host in a group of temporary ectoparasites associating with a range of mammals and birds.

## Supplementary Material

Table S1. List of *Cimex* specimens included in analyses.

## Supplementary Material

Table S2. Information on primers used for amplification of target genes in our study.

## Supplementary Material

Figure S1. Hypothesized species tree of *Cimex spp.* specimens, based on *CO1* and *EF1α*, constructed with *BEAST 2.4.2.

## Data accessibility

All nucleotide sequences obtained in this study can be found on NCBI's Genbank under accession numbers KY561671-KY561757.
