# Supplementary material for: Host association and selection on salivary protein genes in bed bugs and related blood-feeding ectoparasites
Source: R Soc Open Sci. 2017 Jun 21;4(6):170446. doi: 10.1098/rsos.170446 (PMC5493930; doi:10.1098/rsos.170446)
Supplement: Table S1. List of Cimex specimens included in analyses. [file rsos170446supp1.docx]

**Table S1.** List of *Cimex* specimens included in analyses. Host refers to the species with which the ectoparasite was found associated, either being on its body or in a roost or housing unit inhabited mainly by the species. Accession numbers for each of the four genes analysed in the study are listed, except when no amplification could be obtained (indicated with “No amplification”).

| **Sample name** | **Species** | **Host** | **Country** | ***CO1* accession number** | ***EF1α* accession number** | **Apyrase accession number** | **Nitrophorin accession number** | |
| --- | --- | --- | --- | --- | --- | --- | --- | --- |
| 12 | *Cimex lectularius* | *Homo sapiens* (Human) | Canada | KY561671 | KY561686 | KY561709 | KY561735 | |
| 411 | *Cimex lectularius* | *Homo sapiens* (Human) | Canada | KY561672 | KY561687 | KY561710 | KY561736 | |
| 720 | *Cimex lectularius* | *Homo sapiens* (Human) | Austria | KY561673 | KY561688 | KY561711 | KY561737 | |
| 754 | *Cimex lectularius* | *Homo sapiens* (Human) | Italy | KY561674 | KY561689 | KY561712 | KY561738 | |
| 790 | *Cimex lectularius* | *Homo sapiens* (Human) | Finland | KY561675 | KY561690 | KY561713 | KY561739 | |
| 51 | *Cimex lectularius* | *Myotis myotis* (Bat) | Czech Republic | KY561676 | KY561691 | KY561714 | KY561740 | |
| 173 | *Cimex lectularius* | *Myotis myotis* (Bat) | Czech Republic | KJ937980 | KY561692 | KY561715 | KY561741 | |
| 148 | *Cimex hemipterus* | *Homo sapiens* (Human) | Indonesia | KY561677 | KY561693 | KY561716 | KY561742 | |
| 348 | *Cimex hemipterus* | *Homo sapiens* (Human) | Malaysia | KY561678 | KY561694 | KY561717 | KY561743 | |
| RG03 | *Cimex adjunctus* | *Eptesicus fuscus* (Bat) | USA | KU534930 | KY561695 | KY561718 | KY561744 | |
| EFS40 | *Cimex adjunctus* | *Eptesicus fuscus* (Bat) | USA | KU534918 | KY561696 | KY561719 | KY561745 | |
| S1 | *Cimex adjunctus* | *Myotis lucifugus* (Bat) | Canada | KU534929 | KY561697 | KY561720 | KY561746 |  |
| N1 | *Cimex adjunctus* | *Myotis lucifugus* (Bat) | Canada | KU534908 | KY561698 | KY561721 | KY561747 |  |
| 1725 | *Cimex adjunctus* | *Myotis septentrionalis* (Bat) | Canada | KU534906 | KY561699 | KY561722 | KY561748 |  |
| O9 | *Cimex brevis* | *Lasionycteris noctivagans* (Bat) | Canada | KY561679 | KY561700 | KY561723 | KY561749 |  |
| N5 | *Cimex brevis* | *Myotis lucifugus* (Bat) | Canada | KY561680 | KY561701 | KY561724 | KY561750 |  |
| 7193 | *Cimex brevis* | *Myotis lucifugus* (Bat) | Canada | KY561681 | KY561702 | KY561725 | KY561751 |  |
| A5 | *Cimex latipennis* | *Myotis ciliolabrum* (Bat) | Canada | KY561682 | KY561703 | KY561726 | KY561752 |  |
| 61 | *Cimex pipistrelli* | *Myotis myotis* (Bat) | Czech Republic | GU985529 | KY561704 | KY561727 | No amplification |  |
| 108 | *Cimex pipistrelli* | *Myotis daubentoni* (Bat) | Czech Republic | KY561683 | KY561705 | KY561728 | KY561753 |  |
| 350 | *Cimex japonicus* | *Verpertilio superans* (Bat) | Japan | KC503541 | KF018744 | KY561729 | KY561754 |  |
| 351 | *Cimex japonicus* | *Verpertilio superans* (Bat) | Japan | KY561684 | KY561706 | KY561730 | KY561755 |  |
| 149 | *Cimex vicarius* | *Petrochelidon pyrrhonota* (Swallow) | USA | GU985541 | KF018738 | KY561731 | No amplification |  |
| 120 | *Cimex hirundinis* | *Delichon urbica* (Swallow) | Czech Republic | GU985543 | KF018736 | KY561732 | KY561756 |  |
| 895 | *Cimex sp.* | *Delichon dasypus* (Swallow) | Japan | GU985542 | KY561707 | KY561733 | No amplification |  |
| 896 | *Cimex sp.* | *Delichon dasypus* (Swallow) | Japan | KY561685 | KY561708 | KY561734 | KY561757 |  |
