# Supplementary material for: Host association and selection on salivary protein genes in bed bugs and related blood-feeding ectoparasites
Source: R Soc Open Sci. 2017 Jun 21;4(6):170446. doi: 10.1098/rsos.170446 (PMC5493930; doi:10.1098/rsos.170446)
Supplement: Table S2. Information on primers used for amplification of target genes in our study. [file rsos170446supp2.docx]

**Table S2.** Information on primers used for amplification of target genes in our study. Length of target fragment, authors who first introduced them, and optimal Polymerase Chain Reaction annealing temperature are shown.

| **Gene** | **Length of fragment** | **Name of primer** | **Sequence from 5’ to 3’** | **Authors** | **Annealing temperature** |
| --- | --- | --- | --- | --- | --- |
| Cytochrome Oxidase 1  (*CO1*) | 576 bp | Lep1Fdeg | F: ATTCAACCAATCATAAAGATATNGG | Balvín *et al.* 2012 | 42°C |
|  |  | Lep1Rdeg | R: TAWACTTCWGGRTGTCCRAARAATCA |  |  |
|  |  | CO1adj1F | F: TATGAGCAGGCATGTTAGGG | Talbot *et al.* 2016 | 49°C |
|  |  | CO1adf1R | R: ATAGATGTTGATAAAGAATTGGG |  |  |
| Elongation Factor 1 α  (*EF1α*) | 509 bp | Shirley | F: GCYTCGTGGTGCATYTCSAC | Balvín *et al.* 2015 | 57°C |
|  |  | Prowler | R: CAGGCTGATTGYGCTGTACTTATYCTTGC |  |  |
| Apyrase | 369 bp | CadjA1F | F: GTCCAGCCGACGAATGTAGT | Current study | 55°C |
|  |  | CadjA1R | R: CCAGTCCTGTTCTGTCATCG |  |  |
|  |  | A2F | F: CCAGCCGACGAATGTAGTTG | Current study | 56°C |
|  |  | A3R | R: TTACCATCTCCGTCAGCCAG |  |  |
| Nitrophorin | 297 bp | N2F | F: CGATCAGAAGAAATCAGGCGG | Current study | 53°C |
|  |  | N2R3R | R: AGGTAGGCTTGAAGGTGACC |  |  |
