# Supplementary material for: Host association and selection on salivary protein genes in bed bugs and related blood-feeding ectoparasites
Source: R Soc Open Sci. 2017 Jun 21;4(6):170446. doi: 10.1098/rsos.170446 (PMC5493930; doi:10.1098/rsos.170446)
Supplement: Figure S1. Hypothesized species tree of Cimex spp. specimens, based on CO1 and EF1α, constructed with *BEAST 2.4.2. [file rsos170446supp3.pdf]

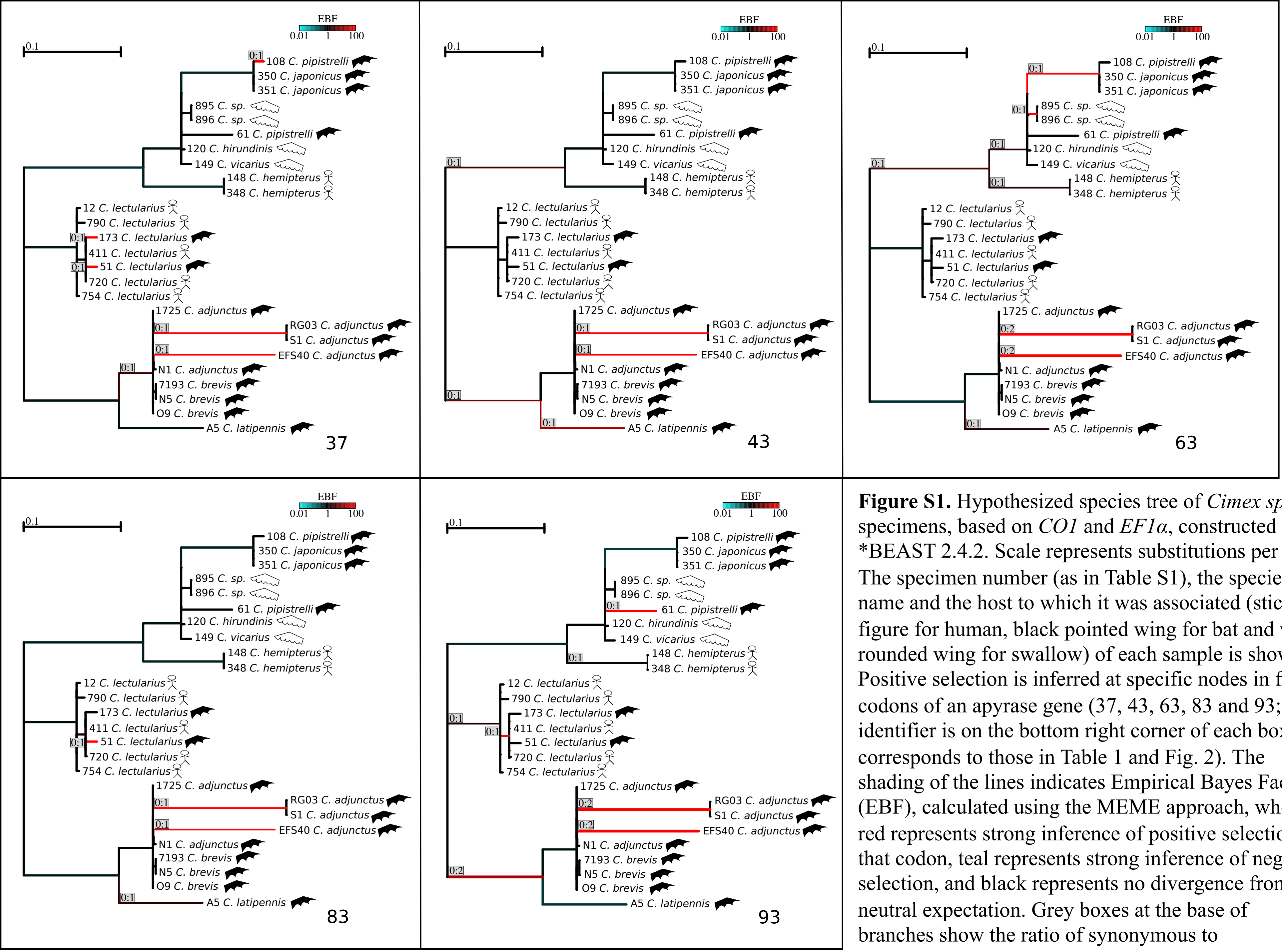

Figure S1. Hypothesized species tree of *Cimex spp.* specimens, based on *CO1* and *EF1α*, constructed with *BEAST 2.4.2. Scale represents substitutions per site. The specimen number (as in Table S1), the species name and the host to which it was associated (stick figure for human, black pointed wing for bat and white rounded wing for swallow) of each sample is shown. Positive selection is inferred at specific nodes in five codons of an apyrase gene (37, 43, 63, 83 and 93; identifier is on the bottom right corner of each box and corresponds to those in Table 1 and Fig. 2). The shading of the lines indicates Empirical Bayes Factor (EBF), calculated using the MEME approach, where red represents strong inference of positive selection at that codon, teal represents strong inference of negative selection, and black represents no divergence from neutral expectation. Grey boxes at the base of branches show the ratio of synonymous to nonsynonymous substitutions at the codon for the respective node.
